# Changes in food intake patterns during 2000–2007 and 2008–2016 in the population-based Northern Sweden Diet Database

**DOI:** 10.1186/s12937-019-0464-0

**Published:** 2019-07-12

**Authors:** Ena Huseinovic, Agneta Hörnell, Ingegerd Johansson, Anders Esberg, Bernt Lindahl, Anna Winkvist

**Affiliations:** 1 Department of Internal Medicine and Clinical Nutrition, the Sahlgrenska Academy, University of Gothenburg, Box 459, SE-405 30 Gothenburg, Sweden; 2 Department of Food and Nutrition, Umeå University, Umeå, Sweden; 3 Department of Odontology, Umeå University, Umeå, Sweden; 4 Department of Public Health and Clinical Medicine, Section of Sustainable Health, Umeå University, Umeå, Sweden

**Keywords:** Food intake patterns, Dietary patterns, FFQ, NSDD, Diet, Time trends

## Abstract

**Background:**

Food intake patterns provide a summary of dietary intake. Few studies have examined trends in food intake patterns over time in large, population-based studies. We examined food intake patterns and related sociodemographic and individual characteristics in the large Northern Sweden Diet Database during the two time windows 2000–2007 and 2008–2016.

**Methods:**

In total, 100 507 participants (51% women) who had filled in a 64-item food frequency questionnaire and provided background and sociodemographic data between 2000 and 2016 were included. Food intake patterns were evaluated for women and men separately for the two time windows 2000–2007 and 2008–2016, respectively. Latent class analysis was used to identify distinct, latent clusters based on 40 food groups.

**Results:**

Among both women and men, a greater proportion of participants were classified into food intake patterns characterized by high-fat spread and high-fat dairy during 2008–2016 compared to 2000–2007. In the earlier time window, these high-fat clusters were related to lower educational level and smoking. Simultaneously, the proportion of women and men classified into a cluster characterized by high intake of fruit, vegetables, and fibre decreased from the earlier to the later time window.

**Conclusion:**

From a public health perspective, the increase in clusters with a high conditional mean for high-fat spread and high-fat dairy and decrease in clusters with a high conditional mean for fruit and vegetables, during the time period 2008–2016 compared to 2000–2007, is worrisome as it indicates a shift away from the recommended food habits. Subgroups of women and men with less healthy dietary patterns in the time window 2008–2016 with lower education, lower age, higher body mass index, lower levels of physical activity and more smoking were identified and future interventions may be targeted towards these groups.

**Electronic supplementary material:**

The online version of this article (10.1186/s12937-019-0464-0) contains supplementary material, which is available to authorized users.

## Background

The Global Burden of Disease project annually provides data to quantify trends in health losses from hundreds of diseases and risk factors in 195 countries. The most recent update [1], found that in 2017 dietary risks were responsible for 11 million deaths and 255 million disability-adjusted life years (DALYs) globally. Dietary improvements could likely prevent one in every five deaths globally, and sub-optimal intakes of whole grains, fruits and sodium were reported to account for more than 50% of deaths and 66% of DALYs attributable to diet. Targeted interventions to improve dietary intake in populations is thus of high priority worldwide.

A large body of evidence identifies dietary habits of importance for maintaining good health [2–4]. Such healthy diets are rich in vegetables, pulses, fruit and berries, nuts and seeds, whole grain, fish and seafood, vegetable oil, and low-fat dairy. In addition to beneficial macro- and micronutrient content of these foods, they also provide several potentially bioactive components such as antioxidants, phenolic compounds, and phytoestrogens that may reduce the risk of non-communicable diseases. In contrast, diets characterized by high intake of red and processed meat, added sugars, fat, and salt have been associated with adverse health effects [2]. Understanding these dietary compounds and their specific framing within whole diets can help develop targeted interventions to improve health at the population level, not just by reducing consumption of unhealthy foods, but also by encouraging people to eat more of health-promoting foods.

In line with these insights, the 5th edition of the Nordic Nutrition Recommendations (NNR) [2], as well as other nutrition recommendations (e.g [5]), that are used in planning diets for various populations have shifted focus from intake of single nutrients to the role of the whole diet, dietary patterns, and specific food groups. To decrease energy density, increase micronutrient density and improve carbohydrate and fat quality, NNR emphasize increased intake of fibre-rich plant foods, fish and seafood, nuts and seed, and vegetable oils and spreads, concurrent with decreased intake of sugar, high-fat dairy products, processed and red meat, and salt [2]. In practice, the advice provided based on the 5th edition of the NNR are similar to the advice based on previous editions.

Further work is now needed to identify pockets in the population with sub-optimal dietary patterns so that interventions can be targeted towards the correct population strata. Here, large population-based studies on dietary intake are needed that allow for comprehensive food pattern analyses. Unfortunately, few such population-based studies with detailed information on dietary intake and health exist, especially where diet has been monitored over time using the same methodology. Most often, Principal Component Analysis (PCA) has been used for generating dietary patterns in nutrition research. This is a “variable-centered” approach that identifies patterns among variables in the data set. A complementary approach is the person-centered, which identifies groups of people who have dietary behaviours in common. This may be more useful for later identifying at risk population groups. An example is here cluster analysis. A more recent technique is Latent Class Analysis (LCA), which has a number of statistical advantages over the more commonly used cluster and principal component analyses.

The Northern Sweden Diet Database (NSDD) [6], is a uniform database integrating self-reported questionnaire data on diet from several research projects in northern Sweden; the largest being the Västerbotten Intervention Project (VIP). NSDD is one of the largest population-based databases within a country in Europe, having monitored dietary intake among over 120 000 individuals with the same methodology since 1985. Using this database, we have previously reported food intake patterns among women and men in northern Sweden during the period 1992–2005 [7]. The aim of the current study was to evaluate associations between diet and health up to today and describe changes in dietary patterns over time using LCA. Hence, we examined food intake patterns and related sociodemographic and individual characteristics in the same cohort using data from the two time windows 2000–2007 and 2008–2016.

## Methods

### Study population

For the present analyses, only NSDD participants from VIP were included. The VIP study is an ongoing, population-based prospective study that runs in the county of Västerbotten in northern Sweden. The county has approximately 260 000 inhabitants of which over 120 000 live in the city of Umeå. Participants in VIP who had also provided dietary intake data to the NSDD were eligible for the present study. Since 1985, residents in the county have been invited by their local health center for a medical examination at 40, 50, and 60 years of age. Until 1996, 30-year olds were also invited, but for financial reasons today this only persists in some communities. Over the years, residents may have participated more than once. The average recruitment rate among available participants has been approximately 60%. Only very limited evidence of selection bias in relation to income, age, and unemployment has been reported [8, 9]. Also, no difference has been observed in cancer incidence in the VIP cohort versus in the general population of Västerbotten, indicating a truly population-based cohort [8].

**Fig. 1:**
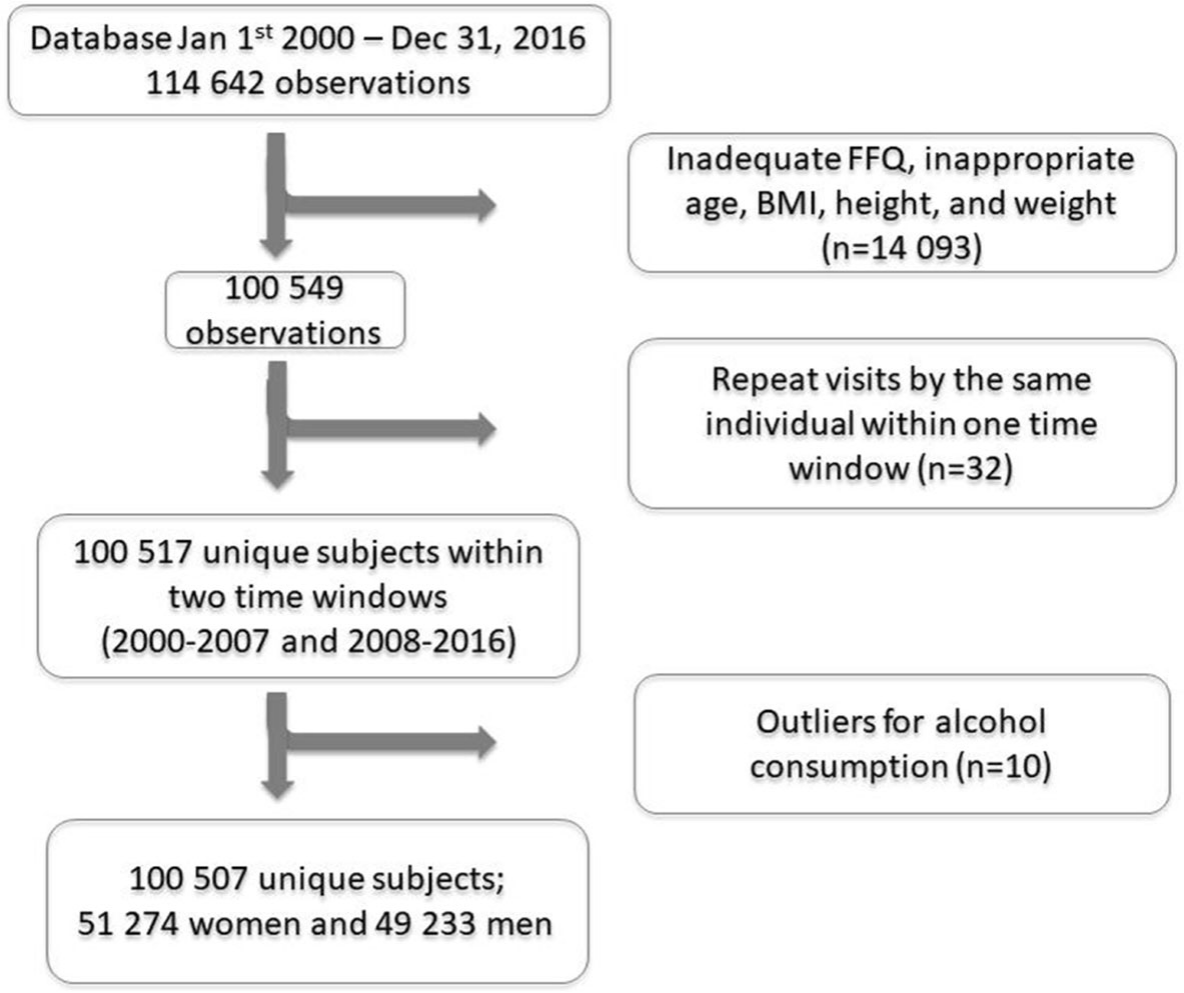
Study flow chart. FFQ, Food Frequency Questionnaire; BMI, Body Mass Index

VIP contained information on 114 642 observations (50.8% women) between January 1st, 2000, to December 31, 2016. Observations were excluded from the present analyses if: food intake recording had ≥ 10% missing data and/or a missing portion indication; food intake recording included 84 items (see below); food intake level (calculated as the estimated total energy intake divided by basal metabolic rate) was extreme (i.e., highest and lowest 1%); energy intake was extreme (lowest 1% and > 5000 kcal); age < 29 years or > 65 years; height < 130 or > 210 cm; weight < 35 kg, or BMI < 15. This reduced the study sample to 100 549 observations (Fig. 1). The data analyses in the present study were cross-sectional despite the longitudinal design of VIP.

Food intake patterns were analysed in two time windows for each gender: between 2000 and 2007 and 2008–2016, respectively. The cut point was chosen because it divided the overall period in two windows of equal size and also because around the years 2004–2007 a heated debate existed in media about low carbohydrate-high-fat diets [10, 11] that likely affected dietary intake trends [12] and possibly the cholesterol levels in the population [13]. Within each gender- and time specific window, repeat visits by the same individual were removed (*n* = 32). In addition, ten outliers for alcohol consumption were removed for women (four in 2000–2007 and six in 2008–2016). The final study sample thus consisted of 100 507 unique subjects. The Research Ethics Committee at Umeå University approved the original study in 1984 (Dnr 2013/332/31) and the Regional Ethics Examination Board in Gothenburg approved the current study in 2017 (Dnr 276–17). Written informed consent was obtained from all participants.

### Diet measurements

At the health visit, participants undergo an extensive health examination, including anthropometric measurements, blood pressure, serum lipid profiles, and an abbreviated oral glucose tolerance test. Also, participants answer an extensive questionnaire on lifestyle, health and life conditions, including a semi-quantitative Food Frequency Questionnaire (FFQ) that covers the preceding year [4]. Initially, the FFQ included 84 food items but from 1996 a shortened version of 64–66 food items was implemented. This reduction was achieved by deleting a few foods that were less common or by merging related food items. For the current analyses, only participants with the shorter FFQ versions were eligible.

In the FFQ, frequency of intake is reported on a 9-level scale. Mealtime portion sizes are estimated with the support of four colour pictures of a plate containing increasing amounts of staple foods (potato/rice/pasta), main protein sources (meat/fish), and vegetables. For other foods, either gender- and age-specific portion sizes or fixed sizes (such as an apple or egg) are applied. Total estimated daily intake of energy (excluding energy from alcohol) and nutrients are calculated by weighting reported intake frequencies by food composition provided by the National Food Agency. Estimated intake of energy, nutrients, vitamins, and minerals has been validated against repeated 24-h dietary records [14, 15] and by serum biomarkers [16, 17] and found to have validity comparable to other FFQs used in cohort studies.

### Demographic, lifestyle and health variables

Background characteristics were collected using detailed self-administered questionnaires including marital status (married/cohabiting and unmarried/other), education (secondary school and less, or academic education), and smoking (smoker, former smoker, and never smoker). Physical activity was measured using the Cambridge index of Physical Activity [18]. This is a validated index based on one question on occupational physical activity and one question on leisure time physical activity. Participants were categorized into inactive (sedentary job and no recreational activity), moderately inactive (sedentary job with < 0.5 h recreational activity per day, *or* standing job with no recreational activity), moderately active (sedentary job with 0.1–1 h recreational activity per day *or* standing job with 0.5 h recreational activity per day *or* physical job with no recreational activity), and active (sedentary job with > 1 h recreational activity per day *or* standing job with > 0.5 h recreational activity per day *or* physical job with at least some recreational activity *or* heavy manual job). Height was measured standing without shoes to the nearest cm. Weight was measured in light clothing to the nearest kg. BMI was calculated as weight (kg) divided by the square of height (m).

### Statistical analyses

Food intake patterns were evaluated for the four different gender- and time specific data sets using LCA in Latent Gold 5.1 [19]. Similar to traditional cluster analysis, LCA groups individuals into mutually exclusive groups by minimizing the within-class variance and maximizing the between-class variance. However, instead of using the K-means algorithm and Euclidean distance to group individuals, LCA utilizes maximum likelihood algorithms and probabilities to identify distinct latent patterns based on a set of observed indicators (variables). Some of the advantages of LCA include the possibility to include indicators of different scale type in the model, provision of more formal criteria to determine the number of classes, 16 embedded random sets of start values, and no need to standardize indicators before analysis [20, 21]. The method has previously been used to examine patterns of temporal eating [22, 23] and weight loss maintenance strategies [24].

For the present analysis, dietary data based on frequency of intake were used as indicators in the analysis. First, food items from the FFQ were grouped into 40 meaningful food groups according to nutrient content and/or culturally relevant culinary preferences (Table 1). To improve robustness of the analyses, frequency of intake was energy-adjusted by using frequency per 1000 kcal. Thereafter, the food groups were entered as continuous indicators in Latent Gold 5.1 and conditional means were interpreted to determine distinguishing food groups within each class. The conditional mean reports the average number of times per day a respondent from latent class k consumes that food group. We initially explored the model fit of 1–7 latent classes by comparing the class solutions using the Bayesian information criteria (BIC), the LL statistics, class size, and pattern interpretability. After reaching the final model, differences in background characteristics and sociodemographics across the classes (or clusters as we refer to them in the remaining text) were examined using ANOVA and Chi-square test in SPSS version 25.0 (Armonk, NY: IBM Corp). For all analyses, *p* < 0.05 was considered statistically significant. Values are presented as mean (SD) and proportions. The study adheres to the STROBE Nut reporting guidelines (Additional file 1).

**Table 1:** Food items included in the 40 food groups used in Latent Cluster Analyses

Food group	Food items
High-fat spreads	Butter and high-fat margarine (80% fat)
Low-fat spreads	Low-fat margarine (40% fat)
Oil	Vegetable oils in cooking and as salad dressing
Butter in cooking	Butter used in cooking
Margarine in cooking	Margarine used in cooking
Fruit	All fruits and berries
High-fiber vegetables	Root vegetables, lettuce, cabbage, kale etc.
Low-fiber vegetables	Tomato, cucumber
Milk, 0.5%	Milk, fermented milk 0.5% fat
Milk, 1.5%	Milk, fermented milk 1.5% fat
Milk, 3.0%	Milk, fermented milk 3.0% fat
Cream	Cream, sour cream, crème fraiche
High-fat cheese	Cheese, hard, 28% fat
Low-fat cheese	Cheese, hard, 17% fat
High-fiber cereals	Oat-, graham- and rye porridge and fiber-rich muesli
Low-fiber cereals	Corn flakes etc.
White bread	White bread, soft and hard
High-fiber bread	High fiber bread, soft and hard
Boiled potato	Boiled and mashed potato
Fried potato	Fried potato and French fries
Pasta and rice	Pasta, macaroni and rice
Fish	High fat and lean fish, shellfish
Red meat	Minced meat, stew, steak
Bacon and sausage	Bacon and sausage
Chicken	Chicken, hen
Cold cuts	Meat, sausage and liverwurst on sandwich
Pancakes and dumplings	Pancakes, dumplings
Pulses	Beans, peas etc.
Sweets	Candies, chocolate
Sugar and jam	Sugar, marmalade, jam, honey
Ice cream	Ice cream
Cookies	Cookies, cakes
Snacks	Chips, popcorn, peanuts
Soda	Sodas
Coffee	Coffee (boiled and filtered)
Tea	Tea
Beer	All types of beer
Wine	Red and white wine
Spirits	All types of spirits
Fast food	Pizza and hamburger

As a complementary analysis of the emerging clusters, PCA and partial least squares (PLS) were run on the final data set to see if matching patterns were detected also with these data driven variable-centered approaches (Simca version 15.0, Umetrics, Sartorius Stedim Biotech, Umeå, Sweden). Input variables were the food groups created for the LCA, expressed as z-scores of frequency of intake per day and per 1000 kcal to obtain standardization and energy adjustment. Factor selection was guided by R^2^ (the explained variation), Q^2^ (the predictive ability), Eigenvalues > 2.0 and interpretability of the factors across samples. Analyses were carried out separately for women and men, and the two time windows, in line with the LCA.

## Results

In total, 24 360 women and 23 107 men were included during 2000–2007 and 26 914 women and 26 126 men during 2008–2016 (Table 2). The mean age was approximately 50 years across all four data sets while BMI was lowest for women during 2000–2007 (mean (SD) 25.8 (4.6) kg/m^2^), and highest for men during 2008–2016 (27.2 (4.1) kg/m^2^). For both women and men, the majority of participants were married/cohabitating (range 78.5–81.5%), had secondary school or less as highest educational level (range 55.7–75.5%), and were non-smokers (range 80.8–87.3%).

**Table 2:** Background characteristics of participants in the Northern Sweden Diet Database during 2000–2007 and 2008–2016

Background characteristic	Women 2000–2007 (*n* = 24 360)	Women 2008–2016 (*n* = 26 914)	Men 2000–2007 (*n* = 23 107)	Men 2008–2016 (*n* = 26 126)
Age^a^ (yrs)	50.1 (8.0)	50.3 (8.2)	50.1 (8.0)	50.2 (8.2)
Height^b^ (cm)	165.1 (6.0)	165.5 (6.2)	178.7 (6.5)	179.3 (6.7)
Weight^b^ (kg)	70.4 (13.1)	71.6 (14.1)	85.4 (13.1)	87.4 (14.4)
Body mass index^b^ (kg/m^2^)	25.8 (4.6)	26.1 (5.0)	26.7 (3.7)	27.2 (4.1)
Marital status^c^ (%)				
Married/cohabitating	81.5	80.7	80.5	78.5
Unmarried/other	18.5	19.3	19.5	21.5
Education (%)				
Secondary school or less	65.4	55.7	75.5	69.5
Academic education	34.6	44.3	24.5	30.5
Physical activity index^d^ (%)				
Inactive	14.9	13.4	16.4	15.3
Moderately inactive	30.7	24.9	30.9	24.3
Moderately active	27.6	27.6	29.3	30.0
Active	26.8	34.1	23.4	30.4
Smoking (%)				
Smoker	19.2	12.7	16.8	13.0
Former smoker	32.5	32.7	33.9	28.6
Never smoker	48.3	54.6	49.3	58.6

Values are mean (SD) and proportions. ^a^Adjusted for year of study participation. ^b^Adjusted for year of study participation and age. ^c^In total, 125, 59, 111, and 78 individuals are missing information on marital status, respectively. ^d^In total, 1593 2180, 1101, and 1900 individuals are missing information on physical activity, respectively

### Latent classes of food intake patterns

**Fig. 2:**
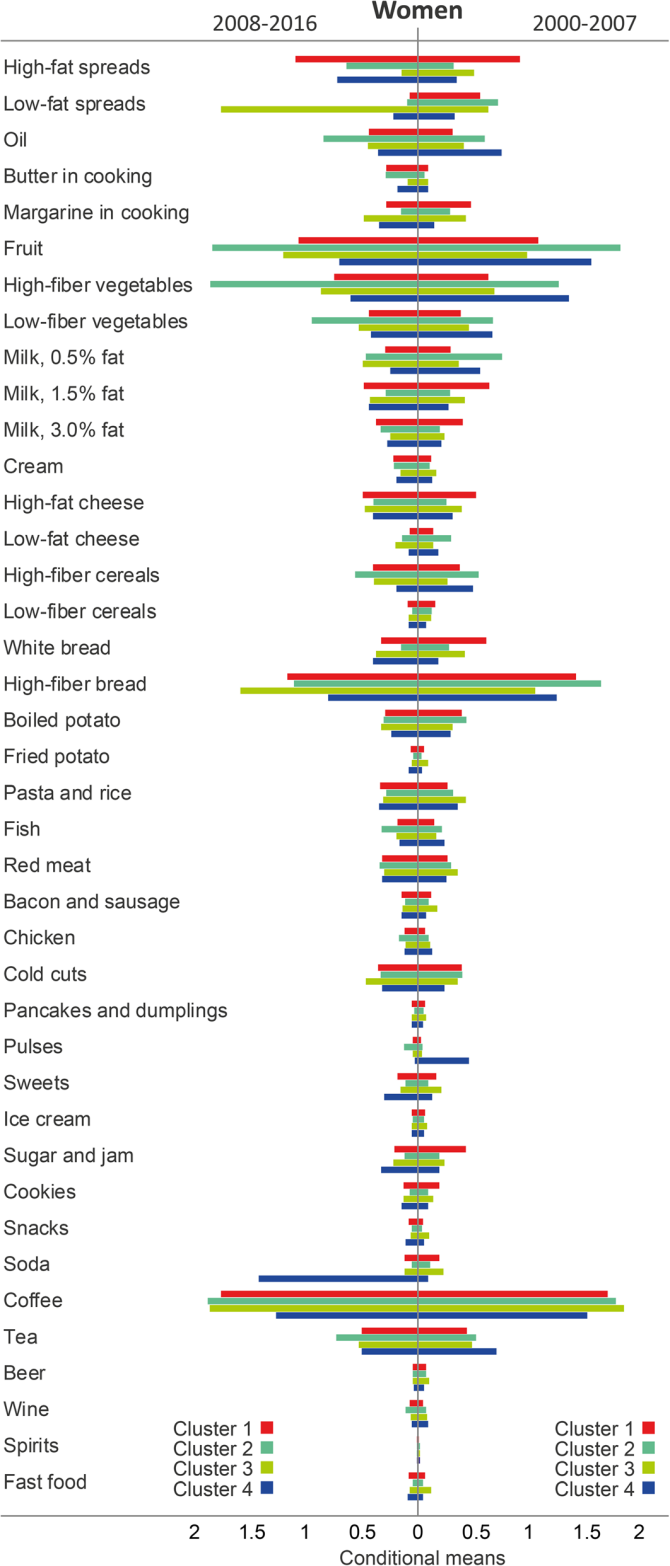
Conditional means demonstrating the differences in response patterns of food groups that distinguish the clusters among women during 2000–2007 and 2008–2016

**Fig. 3:**
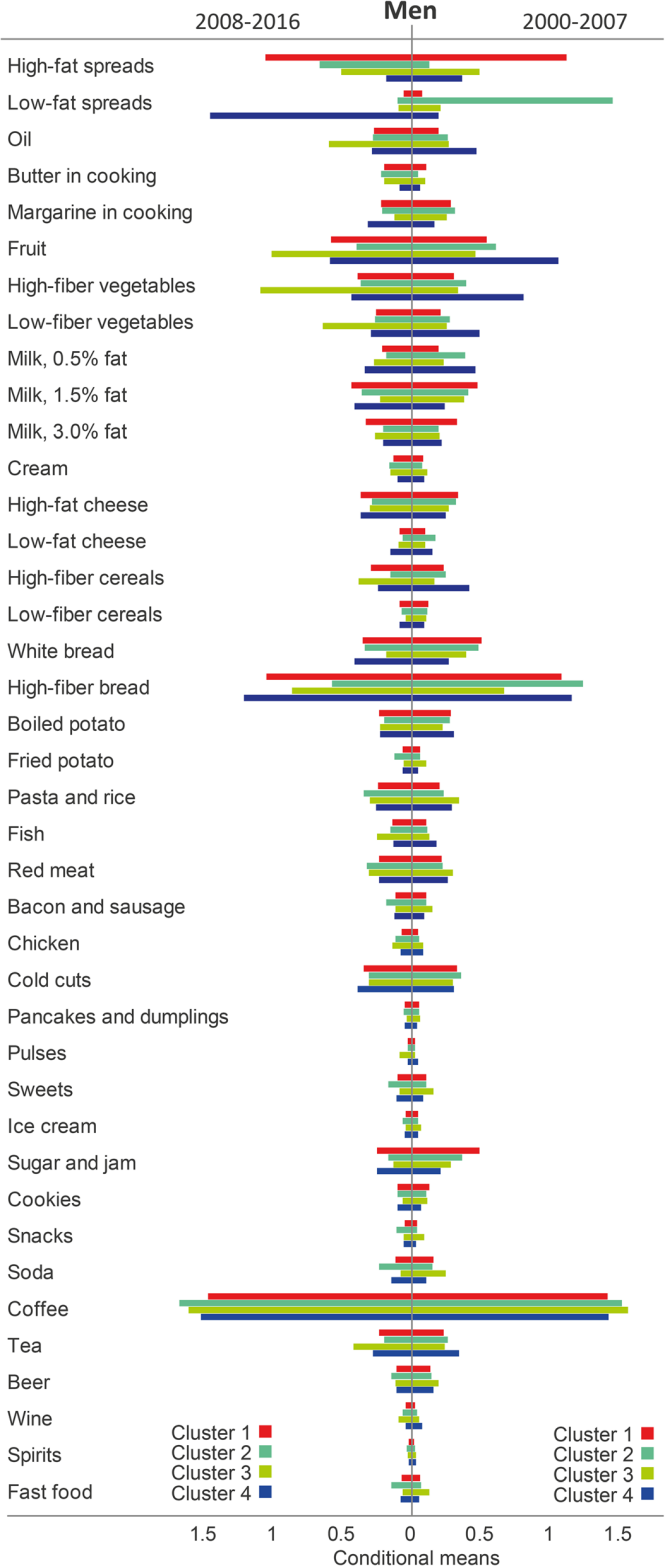
Conditional means demonstrating the differences in response patterns of food groups that distinguish the clusters among men during 2000–2007 and 2008–2016

For all four data sets, model fit indices as well as pattern interpretability favored a four-cluster solution (see Additional file 2: Table S1, for model fit indices). Cluster labels were based on distinguishing features as shown by high conditional means of consuming the different food groups and numbered from largest to smallest group size (Figs. 2 and 3).

### Women during 2000–2007

Among women during 2000–2007, the most common cluster was labeled *High-fat dairy, white bread, sugar/jam and cookies* and comprised 39.7% of the participants in this sex and time window. This cluster had high conditional mean of consuming high-fat spread, full-fat milk, medium-fat milk, high-fat cheese, white bread, sugar/jam, and cookies. The second most common cluster was denoted *Fruit, high-fiber bread and low-fat milk* (35.2% of the participants in this sex and time window) and was characterized by having high conditional mean of consuming fruit, low-fat milk, low-fat cheese, high-fiber grains (i.e., bread and cereal), and low-fat spread. The third cluster, *Bacon/sausage and fast food*, comprised 23.5% of women and was characterized by high conditional mean of consuming bacon/sausage, fast food, snacks, red meat, cream, fried potato, pasta/rice, sweets, and soda. The fourth cluster, which comprised 1.6% of women and was labeled *Pulses and tea*, was characterized by high conditional mean of consuming especially pulses, but also tea and oil. This cluster and the *Fruit, high-fiber bread and low-fat milk* cluster both had high conditional mean of consuming several healthier foods as well as similar low conditional mean of consuming less healthy foods.

The *High-fat dairy, white bread, sugar/jam and cookies* cluster had the lowest proportion of women with academic education (29.0%), and a low, although not the lowest, proportion of participants who reported to be physically active (24.0%) (Table 3). In comparison, 54.8 and 36.6% of women in the *Pulses and tea* class reported to have academic education and to be physically active, respectively. Furthermore, fewer women in the *Fruit, high-fiber bread and low-fat milk* and *Pulses and tea* clusters reported to be current smokers (15.1 and 12.5%, respectively) compared to the other two clusters (21.4 and 22.0%, respectively), all *p* < 0.001.

**Table 3:** Background data among food intake patterns during 2000–2007 for women in the Northern Sweden Diet Database (*n* = 24 360)

	Cluster 1	Cluster 2	Cluster 3	Cluster 4	*P*-value
	High-fat dairy, white bread, sugar/jam and cookies (*n* = 9 670)	Fruit, high-fiber bread and low-fat milk (*n* = 8 579)	Bacon/sausage and fast food (*n* = 5 726)	Pulses and tea (*n* = 385)	
Age (yrs)	50.4 (8.0)	52.7 (7.5)	45.8 (7.1)	50.0 (8.0)	< 0.001
Body weight (kg)	69.7 (13.1)	71.4 (12.9)	70.3 (13.2)	69.4 (13.4)	< 0.001
Body mass index^a^ (kg/m^2^)^a^	25.5 (4.7)	26.3 (4.5)	25.7 (4.6)	25.2 (4.7)	< 0.001
Marital status^a^ (%)					< 0.001
Married/cohabitating	81.5	81.2	82.4	71.7	
Unmarried/other	18.5	18.8	17.6	28.3	
Education (%)					< 0.001
Secondary school or less	71.0	61.4	63.2	45.2	
Academic education	29.0	38.6	36.8	54.8	
Physical activity index^b^ (%)					< 0.001
Inactive	14.5	13.0	18.2	12.9	
Moderately inactive	32.1	29.0	31.3	27.4	
Moderately active	29.4	26.5	26.6	23.1	
Active	24.0	31.5	23.9	36.6	
Smoking (%)					< 0.001
Smoker	21.4	15.1	22.0	12.5	
Former smoker	28.1	37.2	32.6	37.7	
Never smoker	50.5	47.7	45.4	49.9	

Values are mean (SD), and proportions. Continuous variables were analysed using ANOVA and categorical variables were analysed using Chi-square test. ^a^In total, 125 women are missing information on marital status. ^b^In total, 1593 women are missing information on physical activity

### Women during 2008–2016

Among women during 2008–2016, the most common cluster was labeled *High-fat spread and high-fat dairy,* comprised the majority of women in this time window (70.9%) and had high conditional mean of consuming high-fat spread, full-fat milk, medium-fat milk, and high-fat cheese. Women in the second most common cluster, *Fruit, vegetables and oil* (16.9% of the participants), had high conditional mean of consuming fruit, vegetables, oil, high-fiber cereal, fish, chicken, pulses, tea, and wine. The third cluster, *Sandwiches*, comprised 10.4% of women and was characterized by high conditional mean of consuming low-fat spread, high-fiber bread, margarine in cooking, low-fat cheese, and cold cuts. Finally, 1.7% of women were categorized into the *Soda and sweets* cluster which was characterized by high conditional mean of consuming soda, sweets, sugar/jam, snacks, and fried potato.

Compared to the other three clusters, women in the *Soda and sweets* class were younger (47.2 years), had higher BMI (27.2 kg/m^2^), and to a higher degree reported to be unmarried/other (28.1%), physically inactive (18.3%), and current smokers (23.9%). In contrast, women in the *Fruit, vegetables and oil* cluster had the lowest proportion of participants who were physically inactive (9.9%) and current smokers (8.7%). In addition, this cluster had the lowest BMI (26.0 kg/m^2^) and the highest proportion of women with academic education (53.8%), all *p* < 0.001 (Table 4).

**Table 4:** Background data among food intake patterns during 2008–2016 for women in the Northern Sweden Diet Database (*n* = 26 914)

	Cluster 1	Cluster 2	Cluster 3	Cluster 4	*P*-value
	High-fat spread and high-fat dairy (*n* = 19 093)	Fruit, vegetables and oil (*n* = 4 561)	Sandwiches (*n* = 2 792)	Soda and sweets (*n* = 468)	
Age (yrs)	49.5 (8.1)	52.8 (7.7)	52.1 (8.1)	47.2 (7.7)	< 0.001
Body weight (kg)	71.6 (14.2)	70.8 (13.1)	72.6 (14.6)	74.2 (16.4)	< 0.001
Body mass index (kg/m^2^)	26.1 (5.0)	26.0 (4.7)	26.6 (5.2)	27.2 (5.8)	< 0.001
Marital status^a^ (%)					< 0.001
Married/cohabitating	81.3	78.6	81.4	71.9	
Unmarried/other	18.7	21.4	18.6	28.1	
Education (%)					< 0.001
Secondary school or less	56.4	46.2	64.7	66.0	
Academic education	43.6	53.8	35.3	34.0	
Physical activity index^b^ (%)					< 0.001
Inactive	14.2	9.9	13.3	18.3	
Moderately inactive	25.1	21.8	27.6	27.8	
Moderately active	28.4	23.8	28.2	27.1	
Active	32.3	44.4	30.8	26.8	
Smoking (%)					< 0.001
Smoker	13.0	8.7	15.0	23.9	
Former smoker	31.3	38.6	33.7	27.4	
Never smoker	55.6	52.7	51.3	48.7	

Values are mean (SD) and proportions. Continuous variables were analysed using ANOVA and categorical variables were analysed using Chi-square test. ^a^In total, 59 women are missing information on marital status. ^b^In total, 2180 women are missing information on physical activity

### Men during 2000–2007

Among men during 2000–2007, the most common cluster was denoted *High-fat spread, high-fat dairy and sugar/jam,* and comprised 41.6% of the participants in this sex and time window. This cluster had high conditional mean of consuming high-fat spread, full-fat milk, medium-fat milk, and sugar/jam. The *Sandwiches* cluster (23.5% of the participants) had high conditional mean of consuming low-fat spread, margarine in cooking, low-fat cheese, high-fiber bread, and cold cuts. The *Fried potato and fast food* cluster (18.4% of the participants) was characterized by high conditional mean of consuming fried potato, fast food, pasta/rice, red meat, bacon/sausage, cream, sweets, snacks, soda, coffee, and beer. Finally, the *Fruit, vegetables, oil and high-fiber cereals* cluster (16.5% of the participants) showed high conditional mean of fruit, vegetables, oil, low-fat milk, high-fiber cereal, fish, pulses, tea, and wine.

The *Fruit, vegetables, oil and high-fiber cereals* cluster had higher age (52.3 years), lower proportion who were unmarried/other (15.7%) and current smokers (11.5%), and higher proportion who had academic education (39.6%) and were physically active (28.3%) compared to the other three clusters. In contrast, men in the *Fried potato and fast food* cluster were the youngest (45.7 years), while the *High-fat spread, high-fat dairy and sugar/jam* cluster had the highest proportion current smokers (18.9%), all *p* < 0.001, see Table 5.

**Table 5:** Background data among food intake patterns during 2000–2007 for men in the Northern Sweden Diet Database (*n* = 23 107)

	Cluster 1	Cluster 2	Cluster 3	Cluster 4	*P*-value
	High-fat spread, high-fat dairy and sugar/jam (*n* = 9 604)	Sandwiches (*n* = 5 437)	Fried potato and fast food (*n* = 4 260)	Fruit, vegetables, oil and high-fiber cereals (*n* = 3 806)	
Age (yrs)	50.7 (8.0)	50.9 (7.9)	45.7 (6.9)	52.3 (7.7)	< 0.001
Body weight (kg)	84.1 (12.7)	86.6 (13.5)	86.0 (13.2)	86.2 (13.2)	< 0.001
Body mass index (kg/m^2^)	26.3 (3.6)	27.1 (3.8)	26.8 (3.8)	27.0 (3.7)	< 0.001
Marital status^a^ (%)					< 0.001
Married/cohabitating	79.0	82.4	78.2	84.3	
Unmarried/other	21.0	17.6	21.8	15.7	
Education (%)					< 0.001
Secondary school or less	80.6	79.4	72.9	60.4	
Academic education	19.4	20.6	27.1	39.6	
Physical activity index^b^ (%)					< 0.001
Inactive	14.9	17.4	18.0	17.1	
Moderately inactive	31.0	31.5	31.8	28.9	
Moderately active	31.4	28.0	29.3	25.7	
Active	22.7	23.0	20.9	28.3	
Smoking (%)					< 0.001
Smoker	18.9	16.0	18.1	11.5	
Former smoker	32.6	36.6	29.7	38.0	
Never smoker	48.6	47.4	52.2	50.5	

Values are mean (SD) and proportions. Continuous variables were analysed using ANOVA and categorical variables were analysed using Chi-square test. ^a^In total, 111 men are missing information on marital status. ^b^In total, 1101 men are missing information on physical activity

### Men during 2008–2016

Among men during 2008–2016, the most common cluster was labeled *High-fat spread and high-fat dairy* and comprised over half of the participants in this sex and time window (57.1%). This cluster had high conditional mean of consuming high-fat spread, full-fat milk, and medium-fat milk. The second most common cluster, denoted *Fast food, bacon/sausage and fried potato* (21.5% of participants), had high conditional mean of consuming fast food, bacon/sausage, fried potato, pasta/rice, sweets, snacks, soda, coffee, and beer. The two final groups comprised 10.7% each. Members of the *Fruit, vegetables and oil* cluster had high conditional mean of fruit, vegetables, oil, high-fiber cereal, fish, pulses, tea, and wine, while the cluster *Sandwiches* was characterized by low-fat spread, margarine in cooking, low-fat milk, low-fat cheese, white bread, high-fiber bread, and cold cuts.

The *Fruit, vegetables and oil* cluster had the highest proportion men who were married/cohabiting (81.9%), had academic education (47.1%), and were physically active (37.3%) compared to the other three clusters. In contrast, men in the *Fast food, bacon/sausage and fried potato* class were the youngest (46.9 years) and had the highest proportion of current smokers (16.3%), all *p* < 0.001, see Table 6.

**Table 6:** Background data among food intake patterns during 2008–2016 for men in the Northern Sweden Diet Database (*n* = 26 126)

	Cluster 1	Cluster 2	Cluster 3	Cluster 4	*P*-value
	High-fat spread and high-fat dairy (*n* = 14 905)	Fast food, bacon/sausage and fried potato (*n* = 5 629)	Fruit, vegetables and oil (*n* = 2 804)	Sandwiches (*n* = 2 788)	
Age (yrs)	50.9 (8.2)	46.9 (7.4)	51.9 (7.9)	51.6 (8.0)	< 0.001
Body weight (kg)	87.0 (14.0)	88.6 (15.0)	86.8 (14.2)	88.1 (15.1)	< 0.001
Body mass index (kg/m^2^)	27.0 (4.0)	27.6 (4.2)	27.1 (4.0)	27.5 (4.4)	< 0.001
Marital status^a^ (%)					< 0.001
Married/cohabitating	79.5	75.5	81.9	75.9	
Unmarried/other	20.5	24.5	18.1	24.1	
Education (%)					< 0.001
Secondary school or less	71.1	71.1	52.9	74.6	
Academic education	28.9	28.9	47.1	25.4	
Physical activity index^b^ (%)					< 0.001
Inactive	14.2	18.6	14.1	15.4	
Moderately inactive	24.3	25.1	21.2	25.9	
Moderately active	30.1	30.8	27.3	30.2	
Active	31.4	25.5	37.3	28.5	
Smoking (%)					< 0.001
Smoker	12.4	16.3	9.5	13.1	
Former smoker	28.6	25.1	33.7	30.6	
Never smoker	59.1	58.7	56.9	56.3	

Values are mean (SD) and proportions. Continuous variables were analysed using ANOVA and categorical variables were analysed using Chi-square test. ^a^In total, 78 men are missing information on marital status. ^b^In total, 1900 men are missing information on physical activity

### Results from complementary analyses with PCA and PLS

To aid comparability with results from the LCA, score and loading plots were generated with LCA clusters included. For women 2000–2007, good separation was achieved for the first three LCA clusters in components 1 versus 2 (Additional file 3, panel A). The corresponding loadings plot revealed a spectrum on the horizontal axis from healthy (left) to unhealthy (right) foods and on the vertical axis from snacking components (bottom) to meals (top), where again the first three LCA clusters were distinctly separated (Additional file 3, panel C). When displaying components 1 versus 3, the fourth cluster also was separated and this was largely driven by consumption of pulses (Additional file 3, panels B and D, respectively).

For women 2008–2016, scores and loadings plots revealed clear separation for two of the LCA clusters, 1. *High-fat spread and high-fat dairy*; and 3*. Sandwiches* (Additional file 4, panels A and C). Cluster 2, *Fruit, vegetables and oil*, was separated in the loadings plot when comparing components 1 versus 2 (Additional file 4, panel C), and similar to women 2000–2007 spectra were visible on the horizontal axis from healthy (left) to unhealthy (right) foods and on the vertical axis from snacking components (bottom) to meals (top). Cluster 4, *Soda and sweets*, was separated in the loadings plot when comparing components 1 versus 3 and this was largely driven by consumption of soda (Additional file 4, panel D). Still, the scores plot was unable to identify good separation of clusters 2 and 4, even when inspecting components 1 versus 4 (Additional file 4, panel B).

For men 2000–2007, scores and loadings plots of components 1 versus 2 supported LCA results, showing good separation of all four LCA clusters (Additional file 5). Again, loadings spectra were visible on the horizontal axis from healthy (left) to unhealthy (right) foods and on the vertical axis from snacking components (bottom) to meals (top).

For men 2008–2016, scores plot showed separation of all LCA clusters except cluster 1, *High-fat spread and high-fat dairy*, and this cluster was not captured in further components either (Additional file 6, panel A). In corresponding loadings plot, all except cluster 4, S*andwiches*, showed good separation (Additional file 6, panel B). Here, spectra were visible on the horizontal axis from meals (left) to snacking components (right) and on the vertical axis from unhealthy (bottom) to healthy (top) foods. When inspecting loadings of components 1 versus 3, also the fourth cluster was visible and this was largely driven by consumption of low-fat spread (Additional file 6, panel C).

## Discussion

We found that, when the two time windows were compared, both women and men in the latter time window appeared to move away from the Nordic recommendations on food habits [2]. A greater proportion of participants were classified into food intake patterns characterized by high-fat spread and high-fat dairy during 2008–2016 compared to 2000–2007. The increase was more than twice as large among women as among men (+ 79% vs + 37%, respectively). Perhaps this is an indication of a higher propensity for women to adopt new diets [25]. In the earlier time window, but not specifically in the later, these high-fat clusters were related to lower educational level and smoking. Simultaneously, the proportion of women and men classified into clusters characterized by high intake of fruit and vegetables decreased from the earlier to the later time window.

The VIP started in the mid-80s to decrease the prevalence of cardiovascular diseases in Västerbotten County, which then had the highest prevalence in Sweden [26]. The main components of the intervention were to decrease total fat intake, especially through decreased intake of saturated fat, and to increase the physical activity levels. Over the years, several studies have been performed on the growing dataset. One of these used a cross-sectional design to study 25-year time-trends in diet, cholesterol, and BMI between 1986 and 2010 [12]. The shift towards high-fat food patterns in the time-period 2008–2016 in the present study shows a continuation of a shift seen in that study [12]. After an initial decrease in intake of saturated and total fat between 1986 and 1992 among both men and women in the VIP-data, a sharp increase was seen from about 2004, followed by increasing levels of serum cholesterol after 2007 [13]. Although the cross-sectional design of that study made evaluation of causality between dietary fat intake and serum cholesterol levels impossible, the coinciding trends suggest a relationship. Interestingly, the increase in intake of saturated and total fat coincided with the introduction of positive media support for a low carbohydrate-high-fat diet [10] and criticism against official nutrition recommendations [11].

The common features of traditional Nordic dietary habits include a large proportion of milk and dairy products as well as moderate to high consumption of meat and fish, and a moderate intake of fruit and vegetables [2]. It is therefore not surprising that dairy intake was well represented in the largest clusters among women and men in both time segments. The question is whether the intake of saturated fats or the extra calories are of most concern in relation to cardiovascular health when considering high consumption of high-fat dairy and high-fat spreads from dairy. A recent review suggested that bioactive components associated with dairy lipids, especially high fat products, may be beneficial for cardio-metabolic health [27]. In a previous study in the same population, we found that the source of dairy fat, e.g., non-fermented or fermented milk, determined whether a high intake increased or decreased the risk for type 2 diabetes and myocardial infarction [28]. However, generally effect sizes were small.

The present study found relatively similar food patterns in the two time segments; however, the proportion of participants in each cluster showed large shifts. Still, changing food habits is not unique for the NSDD population. Swedish national data among adults from 1997 to 1998 [29] vs 2010–2011 [30] show changes in food habits over time, as well as differences between women and men. Here, intake of vegetables, pulses, fruit, and berries had increased in 2010–2011 compared to 1997–1998, especially among women. Likewise, intake of fish and seafood had increased while intake of potatoes had decreased among both women and men. The changes in food patterns in the present study do not exactly reflect the changes in these national studies. One reason for the discrepancies could be the different data collection methods. In both national studies, prospective food registrations were used, covering 7 days with 2027 participants in 1997–1998 [29], and 4 days with 2797 participants in 2010–2011 [30]. In contrast, the present study covered the habitual intake in the previous 12 months’ food habits using a retrospective FFQ that has remained virtually unchanged since the mid-80’s. Newer food stuffs may thus not be represented.

Using a FFQ with identical questions over an extended time period is a strength as well as a liability in longitudinal nutritional epidemiological studies. Favourably, it allows for correct evaluations of changes in intake of the food stuffs covered. However, if availability of different food items increases in grocery stores and restaurants, a more limited proportion of total food intake will be captured. A decrease in reported total energy intake has been noted in NSDD over a 10-year period when comparing intake data from two consecutive measurements for the same individuals (− 11.5% for women and − 8.8% for men) concurrent with an increase in BMI (+ 4.0% for women and + 3.8% for men) and slight increase in physical activity, indicating a need for further consideration [31].

In the present study, a decreased diversification in food patterns during the latter time period was visible, resulting in one dominating high-fat food pattern for both genders. This may reflect a decreased applicability of the FFQ over time, especially for women where 70.9% belonged in the *High-fat spread and high-fat dairy* cluster. Although the results from the present analysis cannot be directly translated into trends in absolute intake, a decreased applicability of the FFQ may also partly explain the finding of a decreased proportion of participants in clusters characterized by intake of fruit and vegetables, which is contradictory to findings from the national studies [29, 30]. Indeed, there has been large increases in availability of vegetables as well as vegetable-based alternatives to dairy in grocery stores in Sweden, and statistics from the Swedish Board of Agriculture indicate a 25% increase in purchases of vegetables in the Swedish population between 2000 and 2013 [32]. Such a large increase could be expected to be reflected also in the VIP-population. One explanation for the discrepancy could be that national statistics represent changes among the whole population while the present study only includes age groups who may be less inclined to change than younger segments of the population.

In the latest Swedish national dietary study on adults from 2010 to 11 [30], individuals with higher educational level reported higher consumption of fruit, berries, vegetables, cheese, and alcohol compared with individuals with lower educational level. This fits with the present study where groups with a high conditional mean of fruit, vegetables, and wine also had higher educational level in both time-periods for both gender. In both time periods for both sexes, clusters with high conditional mean of fruit and vegetables were associated with higher education, higher levels of physical activity and less smoking, indicating a healthier profile in this population strata. In the later time periods, clusters with high conditional mean of soda and sweets (women) and fast food, bacon/sausage and fried potato (men) were associated with younger age, higher BMI, less physical activity and more smoking, indicating a less healthy profile in this population strata.

The previous study on food patterns in NSDD [7] covered the period 1992–2005. It used cluster analysis and the number and combinations of food groups differed slightly from the present study. Similarities between the two studies include that clusters labelled Fruit and vegetables, and High fat, were identified in both studies and consisted of similar food groups. In the previous study, a cluster *Coffee and sandwich* was identified among women. In the current study, the cluster S*andwiches* was identified in the later time period for both genders. Still, the previous study found a larger variation in frequency of intake among women than among men, resulting in four vs three distinct clusters, respectively. In the present study, both genders had four distinct clusters in both time periods; however the fourth cluster among women was based on one extreme food group and very small in both time periods, including less than 2% of the female population.

### Strengths and limitations of the study

A major strength of the study is the large, population-based sample of Swedish women and men including more than 100 000 individuals between 2000 and 2016. The large sample size permitted separate evaluations of women and men, and of two time periods so that trends over time may be captured. Food intake data were collected using the same methodology throughout the study period, thus permitting evaluations of changes in intake of food groups covered by the FFQ. As mentioned, this may however also imply a limitation in that more recent food stuffs are missed, leading to lower total intake and less variation in intake captured over time.

A novel feature of the study includes the use of LCA to identify latent subgroups within the study population with similar response patterns from the FFQ. Moreover, the study results were strengthened by complementary analyses using the data driven variable-centered methods PCA and PLS. Here, most clusters identified by LCA (except the very smallest) showed separation, and meaningful spectra ranging from healthy to unhealthy foods, and from full meals to snacking components, were visible in the loadings on food groups.

## Conclusion

From a public health perspective, the increase in clusters with a high conditional mean for high-fat spread and high-fat dairy and decrease in clusters with a high conditional mean for fruit and vegetables, during the time period 2008–2016 compared to 2000–2007 is worrisome as it indicates a shift away from the recommended food habits. Subgroups of women and men with less healthy dietary patterns in the time window 2008–2016 with lower education, lower age, higher BMI, lower levels of physical activity and more smoking were identified and future interventions may be targeted towards these groups.

## Additional files

Additional file 1: Adherence of the study according to STROBE-Nut. (DOC 84 kb)

Additional file 2: **Table S1.** Model fit indices for latent class models evaluated for the four data sets in the Northern Sweden Diet Database. (DOCX 13 kb)

Additional file 3: Principal component analysis model displaying separation for women during 2000-2007 related to belonging to the four clusters from Latent Class Analysis (Cluster 1, red. *High-fat dairy, white bread, sugar/jam and cookies*; Cluster 2, dark green. *Fruit, low-fat milk and high-fiber bread*; Cluster 3, light green. *Bacon/sausage and fast food*, and Cluster 4, blue. *Pulses and tea*). Panel A: Scores for components 1 versus 2. Panel B: Scores for components 1 versus 3. Further, loading plots corresponding to the score plots based on the 40 food groups responsible for the variation among the women. Location of the four clusters from Latent Class Analysis are indicated. Panel C: Loadings for components 1 vs 2. Panel D: Loadings for components 1 vs 3. (TIF 3129 kb)

Additional file 4: Principal component analysis model displaying separation for women during 2008-2016 related to belonging to the four classes/clusters from Latent Class Analysis (Cluster1, red. *High-fat spread and high-fat dairy*; Cluster 2, dark green. *Fruit, vegetables and oil*; Cluster 3, light green. *Sandwiches*, and Cluster 4, blue. *Soda and sweets*). Panel A: Scores for components 1 versus 2. Panel B: Scores for components 1 versus 4. Further, loading plots corresponding to the score plots based on the 40 food groups responsible for the variation among the women. Location of the four classes/clusters from Latent Class Analysis are indicated. Panel C: Loadings for components 1 versus 2. Panel D: Loadings for components 1 versus 3. (TIF 5427 kb)

Additional file 5: Principal component analysis model displaying separation for men during 2000-2007 related to belonging to the four clusters from Latent Class Analysis (Cluster 1, red. *High-fat spread, high-fat dairy and sugar/jam*; Cluster 2, dark green. *Sandwiches*; Cluster 3, light green. *Fried potato and fast food*, and Cluster 4, blue. *Fruit, vegetables, oil and high-fiber cereals*). Panel A: Scores for components 1 versus 2. Further, loading plots corresponding to the score plot based on the 40 food groups responsible for the variation among the men. Location of the four clusters from Latent Class Analysis are indicated. Panel B: Loadings for components 1 versus 2. (TIF 4014 kb)

Additional file 6: Principal component analysis model displaying separation for men during 2008-2016 related to belonging to the four clusters from Latent Class Analysis (Cluster 1, red. *High-fat spread and high-fat dairy*; Cluster 2, dark green. *Fast food, bacon/sausage and fried potato*; Cluster 3, light green. *Fruit, vegetables and oil*, and Cluster 4, blue. *Sandwiches*). Panel A. Scores for components 1 versus 2. Further, loading plots corresponding to the score plots based on the 40 food groups responsible for the variation among the men. Location of the four clusters from Latent Class Analysis are indicated. Panel B: Loadings for components 1 versus 2. Panel C: Loadings for components 1 versus 3. (TIF 5241 kb)

## Data Availability

The datasets generated and analyzed during the current study are not publicly available due to Swedish legislations but are available from the Department of Biobank Research, Umeå University, on reasonable request and with appropriate ethical approval and approval of the local expert group. Detailed information is available on the website (https://www.umu.se/en/biobank-research-unit/).

## Abbreviations

BIC: The Bayesian Information Criteria
BMI: Body mass index (kg/m^2^)
DALYs: Disability-adjusted life years
FFQ: Food Frequency Questionnaire
LCA: Latent Class Analysis
NSDD: The Northern Sweden Diet Database
PCA: Principal components analysis
PLS: Partial least squares
VIP: Västerbotten Intervention Project
