# Supplementary material for: Changes in food intake patterns during 2000–2007 and 2008–2016 in the population-based Northern Sweden Diet Database
Source: Nutr J. 2019 Jul 12;18:36. doi: 10.1186/s12937-019-0464-0 (PMC6626352; doi:10.1186/s12937-019-0464-0)
Supplement: Supplementary file 1 — Adherence of the study according to STROBE-Nut. (DOC 84 kb) [file 12937_2019_464_MOESM1_ESM.doc]

STROBE Statement—Checklist of items that should be included in reports of ***cohort studies***

|  | Item No | Recommendation |
| --- | --- | --- |
| **Title and abstract** | 1 | (*a*) The study is a cross-sectional observational study |
|  |  | (*b*) The abstract provides information on what was done and what was found |
| Introduction | | |
| Background/rationale | 2 | Food intake patterns provide a summary of dietary intake. Few studies have examined trends in food intake patterns over time in large, population-based studies. |
| Objectives | 3 | We examined food intake patterns and related individual characteristics in the large Northern Sweden Diet Database (NSDD) during the two time windows 2000-2007 and 2008-2016. |
| Methods | | |
| Study design | 4 | The observational study design together with participant selection are described in the first section of the methods section and specific data in Table 2. |
| Setting | 5 | The study population are women and men (30-70 years of age) in northern Sweden. Recruitments were from 2000 through 2016. The final study group comprised 100 507 observations (51% women and 49% men). |
| Participants | 6 | (*a*) Subjects were eligible if they had participated in the Västerbotten Intervention Programme (VIP) and had a diet recording. Observations were excluded if the food intake recording was incomplete, age <29 years or >65.0 years, and anthropometric measures implausible. |
|  |  | (*b*) No case-control design |
| Variables | 7 | The primary outcome was clusters/classes of food intake. Important covariates included sex, age, marital status, education, physical activity, screening period and smoking. Food items included in the food groups included are shown in Table 1. |
| Data sources/ measurement | 8* | Data were obtained from questionnaires answered at the health screening and from medical assessments performed as part of the health screening and identification of intervention targets. |
| Bias | 9 | The efforts to address potential sources of bias include the large study groups, and adjustment for age and year of study participation in central measures. All analyses are stratified by sex. |
| Study size | 10 | This is a population study where the number of participants was determined by the number of health screening attendants and quality filtering. |
| Quantitative variables | 11 | Quantitative variables were used as continuous measures (as input in cluster analyses) and categorical variables were included as such as covariates. Food intake was expressed per 1000 kcal. |
| Statistical methods | 12 | (*a*) Latent Class Analysis (LCA) and principal component analysis (PCA) for evaluating patterns, and ANOVA and Chi-square test to evaluate differences between clusters/classes. |
|  |  | (*b*) Analyses were performed separate for women and men and also in two time periods. |
|  |  | (*c*) Observations with missing data were excluded. |
|  |  | (*d*) Not applicable |
|  |  | (*e*) Sensitivity analyses included PCA analyses as a second way to inductively create patterns. |
| Results | | |
| Participants | 13* | (a) This is presented in a flow diagram |
|  |  | (b) This is presented in the flow diagram |
|  |  | (c) A flow diagram is used |
| Descriptive data | 14* | (a) This is given in Table 2 |
|  |  | (b) Individuals with missing data were excluded. |
|  |  | (c) Not applicable |
| Outcome data | 15* | Clusters/classes of food patterns are described for both sexes, for two time periods. |
| Main results | 16 | (*a*) Clusters/classes of food patterns are presented as main outcome. Supplement intake has not been included as input data. |
|  |  | (*b*) Not applicable. |
|  |  | (*c*) Not relevant |
| Other analyses | 17 | None beyond what is reported above |
| Discussion | | |
| Key results | 18 | Among both women and men, a greater proportion of participants were classified into food intake patterns characterized by high-fat spread and high-fat dairy during 2008-2016 compared to 2000-2007. In the earlier time window, these high-fat classes were related to lower educational level and smoking. Simultaneously, the proportion of women and men classified into a class characterized by high intake of fruit, vegetables, and fibre decreased from the earlier to the later time window. |
| Limitations | 19 | The limitations are a risk of selection bias and systematic measurement errors in dietary recordings by FFQs. Validation efforts have not found evidence for a systematic selection bias, but confirm underreporting by the FFQ. Group evaluations and the large cohort compensate for random errors to some degree. Finally, food intake data were collected using the same methodology throughout the study period, thus permitting evaluations of changes in intake of food groups covered by the FFQ over time. However, this may however also imply a limitation in that more recent food stuffs are missed, leading to lower total intake and less variation in intake captured over time |
| Interpretation | 20 | The overall interpretation of the results is that there is an increase in intake of high-fat dairy and high-fat spread during the time-period 2008-2016. This is worrisome in a public health perspective, as it indicates a shift away from the Nordic recommendation on food habits. |
| Generalisability | 21 | The results are generalisable to populations with similar settings as the population under study |
| Other information | | |
| Funding | 22 | Swedish Research Council for Health, Working Life and Welfare (FORTE) and Vetenskapsrådet (the Swedish Research Council). |

*Give information separately for exposed and unexposed groups.

**Note:** An Explanation and Elaboration article discusses each checklist item and gives methodological background and published examples of transparent reporting. The STROBE checklist is best used in conjunction with this article (freely available on the Web sites of PLoS Medicine at http://www.plosmedicine.org/, Annals of Internal Medicine at http://www.annals.org/, and Epidemiology at http://www.epidem.com/). Information on the STROBE Initiative is available at http://www.strobe-statement.org.
