# Supplementary material for: Changes in food intake patterns during 2000–2007 and 2008–2016 in the population-based Northern Sweden Diet Database
Source: Nutr J. 2019 Jul 12;18:36. doi: 10.1186/s12937-019-0464-0 (PMC6626352; doi:10.1186/s12937-019-0464-0)
Supplement: Supplementary file 2 — Table S1. Model fit indices for latent class models evaluated for the four data sets in the Northern Sweden Diet Database. (DOCX 13 kb) [file 12937_2019_464_MOESM2_ESM.docx]

|  | **1 class** | **2 classes** | **3 classes** | **4 classes** | **5 classes** | **6 classes** | **7 classes** |
| --- | --- | --- | --- | --- | --- | --- | --- |
| **Women 2000-2007** |  |  |  |  |  |  |  |
| **LL** | 56791.3975 | 70035.7973 | 78687.5180 | 84340.8997 | 89465.8681 | 95266.7603 | 100695,7580 |
| **BIC** | -112774.7393 | -138849.4101 | -155738.7231 | -166631.3578 | -176467.1660 | -187654.8218 | -198098.6886 |
| **Women 2008-2016** |  |  |  |  |  |  |  |
| **LL** | 4145.3255 | 20070.4861 | 31912.9158 | 38850.9662 | 46021.8471 | 50974.1349 | 59175.7569 |
| **BIC** | -7474.6188 | -38906.7235 | -62173.3663 | -75631.2507 | -89554.7961 | -99041.1552 | -115026.1828 |
| **Men 2000-2007** |  |  |  |  |  |  |  |
| **LL** | 276894.4028 | 288333.0769 | 295104.1622 | 303519.3597 | 307846.7358 | 312740.3124 | 316480.0813 |
| **BIC** | -552984.9744 | -575450.3591 | -588580.5661 | -604998.9975 | -613241.7863 | -622616.9760 | -629684.5502 |
| **Men 2008-2016** |  |  |  |  |  |  |  |
| **LL** | 270592.3930 | 285402.0383 | 296040.4219 | 298141.7242 | 309789.3868 | 313863.4802 | 321192.4362 |
| **BIC** | -540371.1312 | -569573.4236 | -590433.1927 | -594218.7992 | -617097.1262 | -624828.3148 | -639069.2287 |

**Supplementary table S1. Model fit indices for latent class models evaluated for the four data sets in the Northern Sweden Diet Database.**

LL: log-likelihood, BIC: Bayesian Information Criterion.
