# Supplementary figures and images for: Changes in food intake patterns during 2000–2007 and 2008–2016 in the population-based Northern Sweden Diet Database
Source: Nutr J. 2019 Jul 12;18:36. doi: 10.1186/s12937-019-0464-0 (PMC6626352; doi:10.1186/s12937-019-0464-0)

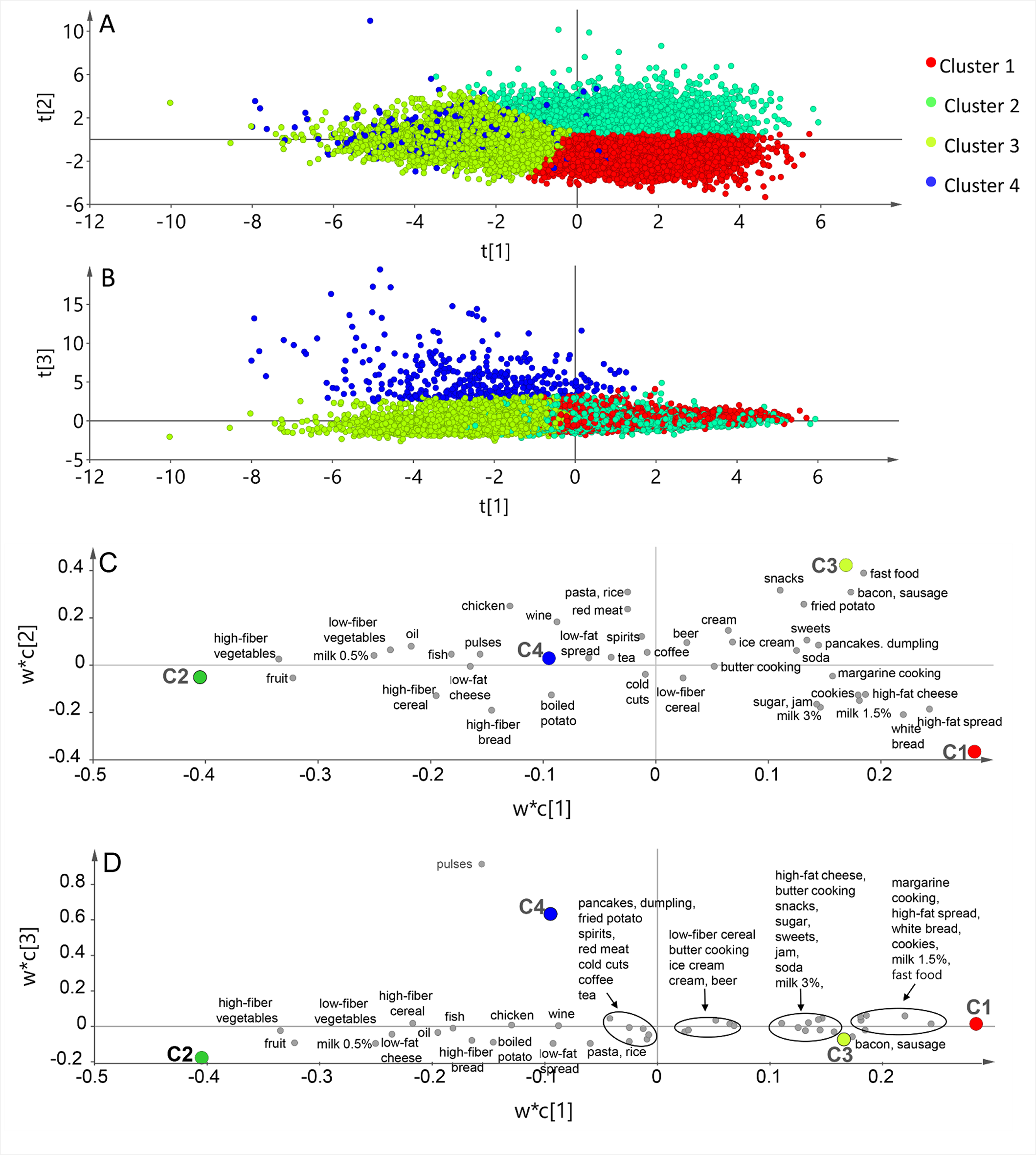

Supplement: Supplementary file 3 — Principal component analysis model displaying separation for women during 2000-2007 related to belonging to the four clusters from Latent Class Analysis (Cluster 1, red. High-fat dairy, white bread, sugar/jam and cookies; Cluster 2, dark green. Fruit, low-fat milk and high-fiber bread; Cluster 3, light green. Bacon/sausage and fast food, and Cluster 4, blue. Pulses and tea). Panel A: Scores for components 1 versus 2. Panel B: Scores for components 1 versus 3. Further, loading plots corresponding to the score plots based on the 40 food groups responsible for the variation among the women. Location of the four clusters from Latent Class Analysis are indicated. Panel C: Loadings for components 1 vs 2. Panel D: Loadings for components 1 vs 3. (TIF 3129 kb) [file 12937_2019_464_MOESM3_ESM.tif]

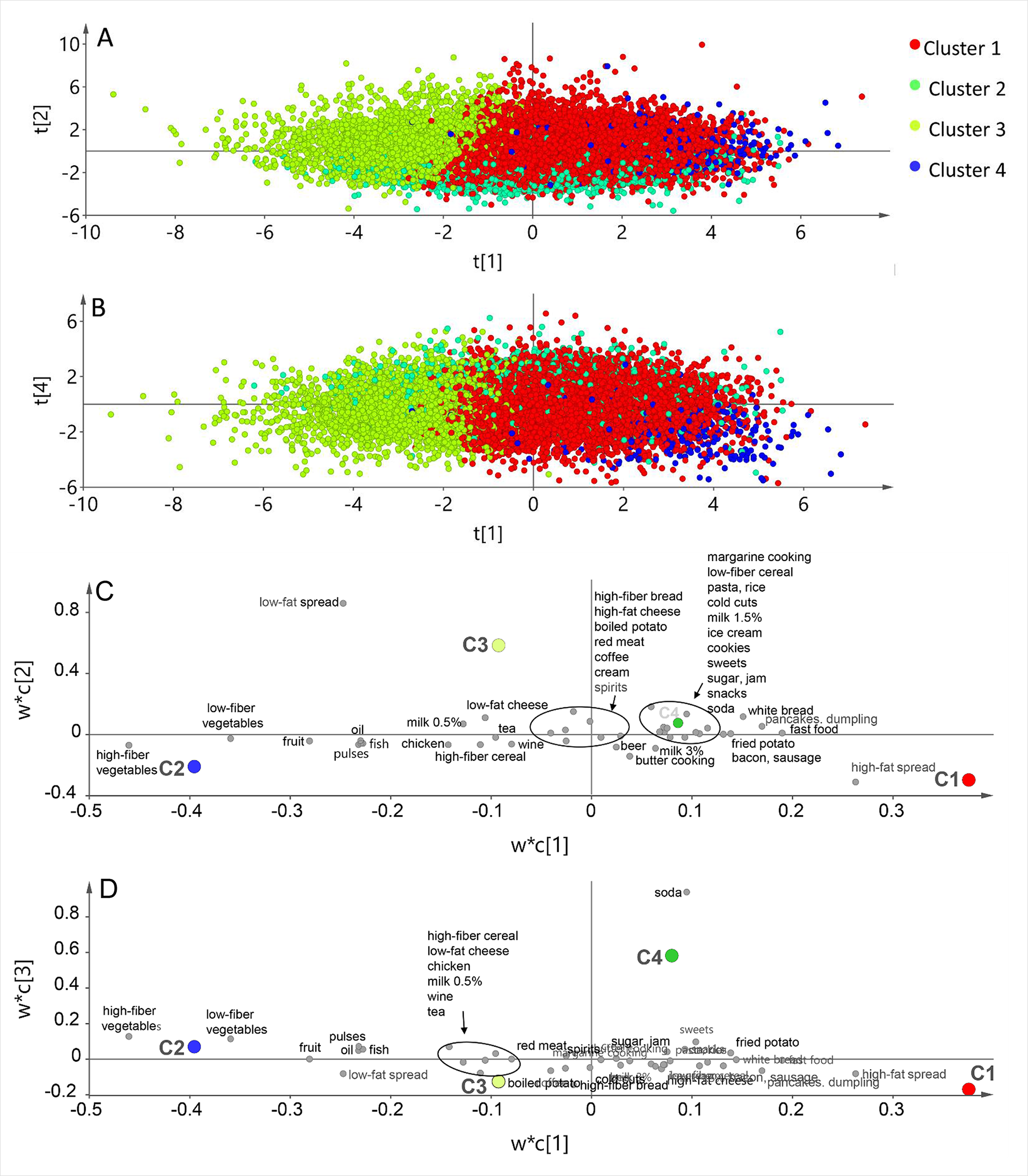

Supplement: Supplementary file 4 — Principal component analysis model displaying separation for women during 2008-2016 related to belonging to the four classes/clusters from Latent Class Analysis (Cluster1, red. High-fat spread and high-fat dairy; Cluster 2, dark green. Fruit, vegetables and oil; Cluster 3, light green. Sandwiches, and Cluster 4, blue. Soda and sweets). Panel A: Scores for components 1 versus 2. Panel B: Scores for components 1 versus 4. Further, loading plots corresponding to the score plots based on the 40 food groups responsible for the variation among the women. Location of the four classes/clusters from Latent Class Analysis are indicated. Panel C: Loadings for components 1 versus 2. Panel D: Loadings for components 1 versus 3. (TIF 5427 kb) [file 12937_2019_464_MOESM4_ESM.tif]

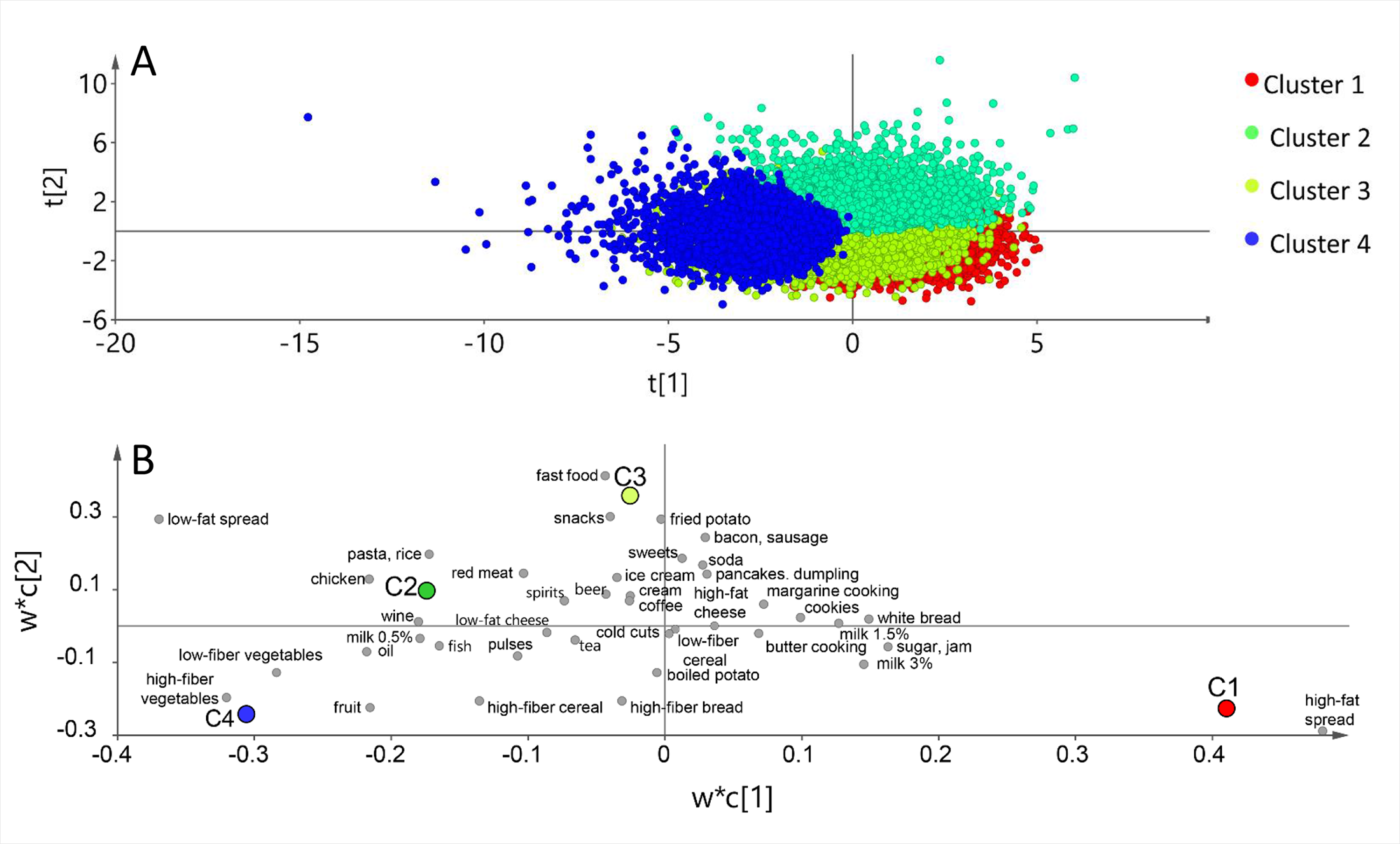

Supplement: Supplementary file 5 — Principal component analysis model displaying separation for men during 2000-2007 related to belonging to the four clusters from Latent Class Analysis (Cluster 1, red. High-fat spread, high-fat dairy and sugar/jam; Cluster 2, dark green. Sandwiches; Cluster 3, light green. Fried potato and fast food, and Cluster 4, blue. Fruit, vegetables, oil and high-fiber cereals). Panel A: Scores for components 1 versus 2. Further, loading plots corresponding to the score plot based on the 40 food groups responsible for the variation among the men. Location of the four clusters from Latent Class Analysis are indicated. Panel B: Loadings for components 1 versus 2. (TIF 4014 kb) [file 12937_2019_464_MOESM5_ESM.tif]

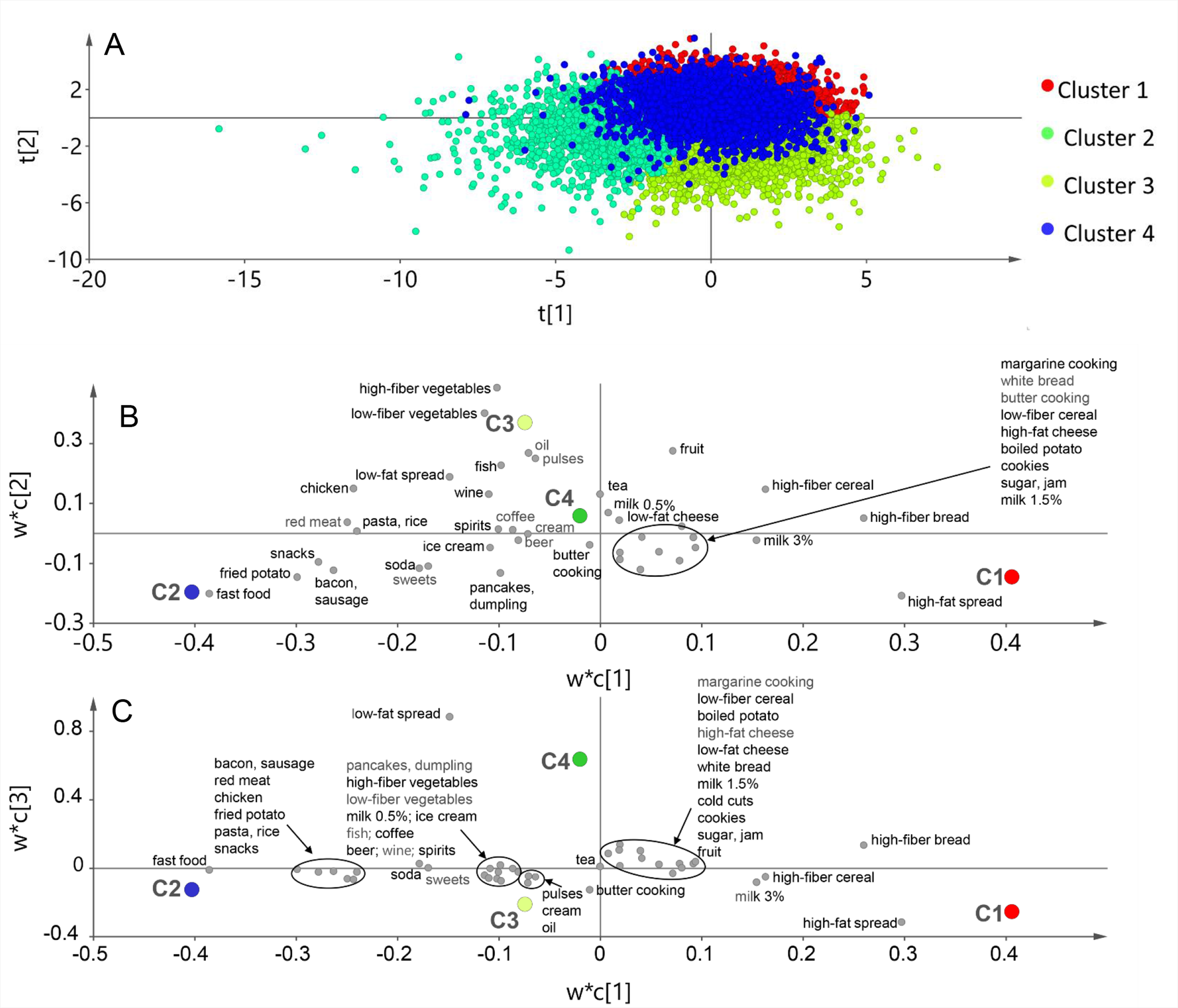

Supplement: Supplementary file 6 — Principal component analysis model displaying separation for men during 2008-2016 related to belonging to the four clusters from Latent Class Analysis (Cluster 1, red. High-fat spread and high-fat dairy; Cluster 2, dark green. Fast food, bacon/sausage and fried potato; Cluster 3, light green. Fruit, vegetables and oil, and Cluster 4, blue. Sandwiches). Panel A. Scores for components 1 versus 2. Further, loading plots corresponding to the score plots based on the 40 food groups responsible for the variation among the men. Location of the four clusters from Latent Class Analysis are indicated. Panel B: Loadings for components 1 versus 2. Panel C: Loadings for components 1 versus 3. (TIF 5241 kb) [file 12937_2019_464_MOESM6_ESM.tif]
